# Big in Japan: Regularizing Networks for Solving Inverse Problems

**DOI:** 10.1007/s10851-019-00911-1

**Published:** 2019-10-03

**Authors:** Johannes Schwab, Stephan Antholzer, Markus Haltmeier

**Affiliations:** Department of Mathematics, University of Innsbruck, Technikerstrasse 13, 6020 Innsbruck, Austria

**Keywords:** Inverse problems, Regularizing networks, Convergence analysis, Convolutional neural networks, Convergence rates, Null space networks, 65J20, 65J22, 45F05

## Abstract

Deep learning and (deep) neural networks are emerging tools to address inverse problems and image reconstruction tasks. Despite outstanding performance, the mathematical analysis for solving inverse problems by neural networks is mostly missing. In this paper, we introduce and rigorously analyze families of deep regularizing neural networks (RegNets) of the form $\mathbf {B}_\alpha + \mathbf {N}_{\theta (\alpha )} \mathbf {B}_\alpha$, where $\mathbf {B}_\alpha$ is a classical regularization and the network $\mathbf {N}_{\theta (\alpha )} \mathbf {B}_\alpha$ is trained to recover the missing part ${\text {Id}}_X - \mathbf {B}_\alpha$ not found by the classical regularization. We show that these regularizing networks yield a convergent regularization method for solving inverse problems. Additionally, we derive convergence rates (quantitative error estimates) assuming a sufficient decay of the associated distance function. We demonstrate that our results recover existing convergence and convergence rates results for filter-based regularization methods as well as the recently introduced null space network as special cases. Numerical results are presented for a tomographic sparse data problem, which clearly demonstrate that the proposed RegNets improve classical regularization as well as the null space network.

## Introduction

This paper is concerned with solving inverse problems of the form

1.1 $$\begin{aligned} y_{\delta }= \mathbf {A}x+ z , \end{aligned}$$

where $\mathbf {A}:\mathbb {X}\rightarrow \mathbb {Y}$ is a bounded linear operator between Hilbert spaces $\mathbb {X}$ and $\mathbb {Y}$, and *z* denotes the data distortion that satisfies ${\left\| z\right\| } \le \delta$ for some noise level $\delta \ge 0$. Many inverse problems arising in medical imaging, signal processing, astronomy, computer vision and other fields are written in form (1.1). A main characteristic property of inverse problems is that they are ill-posed [7, 19]. This means that the solution of (1.1) is either not unique or is unstable with respect to data perturbations.

To solve such kind of inverse problems, one has to employ regularization methods, which serve the following two main purposes:

- Select particular solutions of the noise-free equation, thereby accounting for non-uniqueness $\ker (\mathbf {A}) \ne \{0 \}$.
- Approximate (1.1) by neighboring but stabler problems.

Our aim is finding convergent regularization methods for the solution of (1.1) using deep neural networks that can be adjusted to realistic training data.

In [21], we focused on the non-uniqueness issue, where particular solutions of the noise-free equation, (1.1) with $z=0$, are approximated using classical regularization methods combined with null space networks. Null space networks (introduced originally in [16] in a finite dimensional setting) are refined residual networks, where the residual is projected onto the null space of the operator $\mathbf {A}$. In this context, the stabilization of finding a solution to (1.1) comes from a given traditional regularization method, and the role of the network is to select correct solutions in a data consistent manner.

### Proposed Regularizing Networks (RegNets)

In this paper, we go one step further and generalize the concept of deep null space learning by allowing the network to also act in the orthogonal complement of the null space of $\mathbf {A}$ in a controlled manner. This is in particular useful if the operator contains several small singular values that are not strictly equal to zero. Similar to the components in the kernel, these parts are difficult to be reconstructed by a classical linear regularization method, and quantitative error estimates require strong smoothness assumptions on the objects to be recovered. Learning almost invisible components can significantly improve reconstruction results for less smooth objects.

The proposed RegNets generalize the structure of null space networks analyzed in [21] and consist of a family $(\mathbf {R}_\alpha )_{\alpha >0}$ of mappings $\mathbf {R}_\alpha :\mathbb {Y}\rightarrow \mathbb {X}$ of the form

1.2 $$\begin{aligned} \mathbf {R}_\alpha :=\mathbf {B}_\alpha + \mathbf {N}_{\theta (\alpha )} \mathbf {B}_\alpha \quad \text { for } \alpha >0 . \end{aligned}$$

Here, $(\mathbf {B}_\alpha )_{\alpha >0}$ with $\mathbf {B}_\alpha :\mathbb {Y}\rightarrow \mathbb {X}$ is a classical regularization of the Moore–Penrose inverse $\mathbf {A}^{\varvec{\texttt {+}}}$, and $\mathbf {N}_{\theta (\alpha )} :\mathbb {X}\rightarrow \mathbb {X}$ are neural networks that can be trained to map the part $\mathbf {B}_\alpha \mathbf {A}x$ recovered by the regularization method to the missing part $({\text {Id}}_X-\mathbf {B}_\alpha \mathbf {A}) x$. Here, $(\mathbf {N}_{\theta })_{\theta \in \Theta }$ is any family of parameterized functions that can be taken as a standard network, for example, a convolutional neural network (CNN). In particular, $\mathbf {N}_{\theta (\alpha )}$ is allowed to depend on the regularization parameter $\alpha$.

In this paper, we show that if $\mathbf {N}_{\theta (\alpha )} \mathbf {B}_\alpha \mathbf {A}\rightarrow \mathbf {N}$ on ${\text {ran}}(\mathbf {A}^{\varvec{\texttt {+}}})$ as $\alpha \rightarrow 0$ for some function $\mathbf {N}:\mathbb {X}\rightarrow \mathbb {X}$ with ${\text {ran}}( \mathbf {N})\subseteq \ker (\mathbf {A})$, the RegNets defined by (1.2) yield a convergent regularization method with admissible set $\mathbb {M}:=({\text {Id}}_X + \mathbf {N}) ( {\text {ran}}(\mathbf {A}^{\varvec{\texttt {+}}}) )$. Further, we derive convergence rates (quantitative error estimates) for elements satisfying conditions different from the classical smoothness assumptions.

### Outline

The organization of this paper is as follows. In Sect. 2 we present some background and related results. In Sect. 3, we introduce the proposed regularizing networks and show that they yield a convergent regularization method. Further, we derive convergence rates under a modified source condition. In Sect. 4, we demonstrate that our results contain existing convergence results as special cases. This includes filter-based methods, classical Tikhonov regularization, and regularization by null space networks. Moreover, we examine a data-driven extension of singular components, where the classical regularization method is given by truncated singular value decomposition (SVD). The paper concludes with a short summary presented in Sect. 6.

## Some Background

Before actually analyzing the RegNets, we recall basic notions and concepts from regularization of inverse problems (see [7, 19]) and the concept of null space networks. We also review some previous related work.

### Classical Regularization of Inverse Problems

Regularization methods to stably find a solution of (1.1) use a-priori information about the unknown, for example that the solution $x$ lies in a particular set of admissible elements $\mathbb {M}$. For such a set $\mathbb {M}\subseteq \mathbb {X}$, a regularization method is a tuple $((\mathbf {B}_\alpha )_{\alpha >0},\alpha ^\star )$, where $\mathbf {B}_\alpha :\mathbb {Y}\rightarrow \mathbb {X}$ are continuous operators, and $\alpha ^\star (\delta ,y_{\delta })$ is a parameter choice function such that for all $x\in \mathbb {M}$, we have $\mathbf {B}_{\alpha ^{\star }(\delta ,y_{\delta })}(y_{\delta })\rightarrow x$ as $\delta \rightarrow 0$.

Classical regularization methods approximate the Moore–Penrose inverse $\mathbf {A}^{\varvec{\texttt {+}}}$ and the set $\mathbb {M}$ is given by $\mathbb {M}=\ker (\mathbf {A})^\perp$. Note that for any $y \in {\text {ran}}(\mathbf {A})$, the Moore–Penrose inverse $\mathbf {A}^{\varvec{\texttt {+}}}y$ is given by the minimal norm solution of (1.1). A precise definition of a regularization method is as follows.

#### Definition 1

(*Regularization method*) Let $(\mathbf {B}_\alpha )_{\alpha >0}$ a family of continuous operators $\mathbf {B}_\alpha :\mathbb {Y}\rightarrow \mathbb {X}$ and suppose $\alpha ^\star :(0,\infty )\times \mathbb {Y}\rightarrow (0,\infty )$. The pair $((\mathbf {B}_\alpha )_{\alpha >0},\alpha ^\star )$ is called a (classical) regularization method for the solution of $\mathbf {A}x=y$ with $y\in {\text {dom}}(\mathbf {A}^{\varvec{\texttt {+}}})$, if the following holds

- $$\lim _{\delta \rightarrow 0} \sup \{\alpha ^\star (\delta ,y_{\delta })\mid y_{\delta }\in \mathbb {Y}, \Vert y_{\delta }-y\Vert \le \delta \}=0$$.
- $$\lim _{\delta \rightarrow 0} \sup \{\Vert \mathbf {A}^{\varvec{\texttt {+}}}y-\mathbf {B}_{\alpha ^\star (\delta ,y_{\delta })}y_{\delta }\Vert \mid y_{\delta }\in \mathbb {Y}\text { and } \Vert y_{\delta }-y\Vert \le \delta \}=0$$.

The parameter choice $\alpha ^\star$, depending on the noise level as well as on the data, determines the level of approximation of the Moore–Penrose inverse. For decreasing noise level, the ill-posed problem (1.1) can be approximated by stable problems getting closer to finding the minimum norm solution of (1.1) and in the limit, it holds $\lim _{\delta \rightarrow 0}\mathbf {B}_{\alpha ^\star (\delta ,y_{\delta })}(y_{\delta })=\mathbf {A}^{\varvec{\texttt {+}}}y$.

A great variety of regularization methods, namely filter-based regularization methods, can be defined by regularizing filters.

#### Definition 2

(*Regularizing filter*) A family $(g_\alpha )_{\alpha >0}$ of piecewise continuous functions $g_\alpha :[0,\Vert \mathbf {A}^*\mathbf {A}\Vert ]\rightarrow \mathbb {R}$ is called regularizing filter if

- $$\sup \{|\lambda g_\alpha (\lambda )| \mid \alpha >0 \text { and } \lambda \in [0,\Vert \mathbf {A}^* \mathbf {A}\Vert ]\}< \infty $$.
- $$\forall \lambda \in (0,\Vert \mathbf {A}^* \mathbf {A}\Vert ]:\lim _{\alpha \rightarrow 0} g_\alpha (\lambda )=1/\lambda $$.

Any regularizing filter $(g_\alpha )_{\alpha >0}$ defines a regularization method by taking

2.1 $$\begin{aligned} \forall \alpha >0 :\quad \mathbf {B}_\alpha :=g_\alpha (\mathbf {A}^* \mathbf {A})\mathbf {A}^* . \end{aligned}$$

We call a regularization according to (2.1) a (classical) filter-based regularization. Note that $\mathbf {A}^* \mathbf {A}:\mathbb {X}\rightarrow \mathbb {X}$ is a self-adjoint bounded linear operator, and therefore $g_\alpha (\mathbf {A}^* \mathbf {A}) :\mathbb {X}\rightarrow \mathbb {X}$ is bounded linear as well, defined by the framework of functional calculus [10, 23]. In particular, if $\mathbf {A}^* \mathbf {A}$ has an eigenvalue decomposition $\mathbf {A}^* \mathbf {A}(x) = \sum _{n\in \mathbb {N}} \lambda _n \langle u_n,x\rangle u_n$, then

$$\begin{aligned} \forall x \in \mathbb {X}:\quad g_\alpha (\mathbf {A}^* \mathbf {A}) x:=\sum _{n\in \mathbb {N}} g_\alpha (\lambda _n) \langle u_n, x\rangle u_n . \end{aligned}$$

In the general case, the spectral decomposition of $\mathbf {A}^* \mathbf {A}$ is used to rigorously define $g_\alpha (\mathbf {A}^* \mathbf {A})$, see [10, 23].

**Fig. 1:**
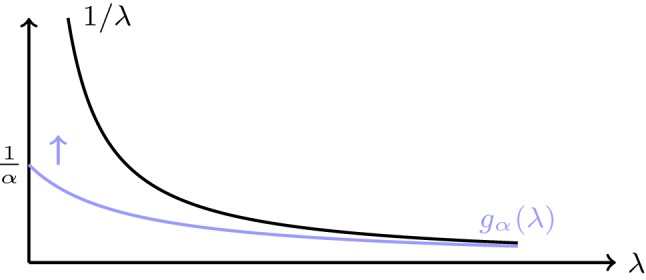
Illustration of the regularizing filter for Tikhonov regularization

**Fig. 2:**
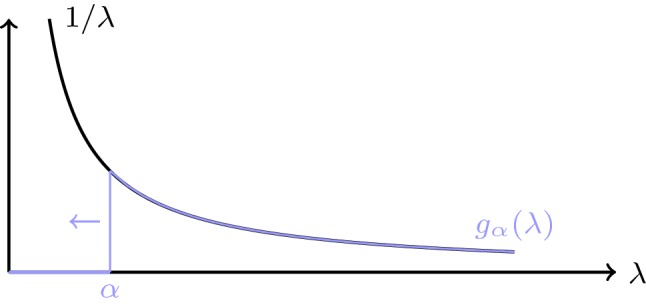
Illustration of the regularizing filter for truncated SVD

Two prominent examples of filter-based regularization methods are classical Tikhonov regularization and truncated SVD. In Tikhonov regularization, the regularizing filter is given by $g_\alpha (\lambda )=1/(\lambda +\alpha )$, see Fig. 1. This yields $\mathbf {B}_\alpha =(\mathbf {A}^*\mathbf {A}+\alpha {\text {Id}}_X)^{-1}\mathbf {A}^*$. In truncated SVD, the regularizing filter is given by

2.2 $$\begin{aligned} g_\alpha (\lambda )={\left\{ \begin{array}{ll} 0, \quad &{}\lambda < \alpha \\ \frac{1}{\lambda } &{}\lambda \ge \alpha , \end{array}\right. } \end{aligned}$$

as shown in Fig. 2. For both methods, the admissible set is $\mathbb {M}=\ker (\mathbf {A})^\perp$.

Other typical filter-based regularization methods are the Landweber iteration and iterative Tikhonov regularization [7].

### Null Space Networks

Standard regularization approximates the Moore–Penrose inverse, and therefore selects elements in $\ker (\mathbf {A})^\perp$. In [21], we introduced regularization with null space networks, where the aim is to approximate elements in a set $\mathbb {M}$ different from $\ker (\mathbf {A})^\perp$.

Null space networks are defined as follows.

#### Definition 3

(*Null space network*) We call a function ${\text {Id}}_X + \mathbf {N}:\mathbb {X}\rightarrow \mathbb {X}$ a null space network if $\mathbf {N}=\mathbf {P}_{\ker (\mathbf {A})} \mathbf {U}$ where $\mathbf {U}:\mathbb {X}\rightarrow \mathbb {X}$ is any Lipschitz continuous function.

Moreover, we use the following generalized notion of a regularization method.

#### Definition 4

(*Regularization methods with admissible set*$\mathbb {M}$) Let $(\mathbf {R}_\alpha )_{\alpha >0}$ be a family of continuous operators $\mathbf {R}_\alpha :\mathbb {Y}\rightarrow \mathbb {X}$ and $\alpha ^\star :(0,\infty )\times \mathbb {Y}\rightarrow (0,\infty )$. Then, the pair $((\mathbf {R}_\alpha )_{\alpha >0},\alpha ^\star )$ is called a regularization method (for the solution of $\mathbf {A}x=y$) with admissible set $\mathbb {M}$, if for all $x\in \mathbb {M}$, it holds

- $$\lim _{\delta \rightarrow 0} \sup \{\alpha ^\star (\delta ,y_{\delta })\mid y_{\delta }\in \mathbb {Y}, \Vert y_\delta - \mathbf {A} x\Vert \le \delta \}=0$$.
- $$\lim _{\delta \rightarrow 0} \sup \{\Vert x -\mathbf {R}_{\alpha ^\star (\delta ,y_{\delta })}y_{\delta }\Vert \mid y_{\delta }\in \mathbb {Y}\text { and } \Vert y_{\delta }-\mathbf {A}x\Vert \le \delta \}=0$$.

In this case, we call $(\mathbf {R}_\alpha )_{\alpha >0}$ an $(\mathbf {A},\mathbb {M})$-regularization.

**Fig. 3:**
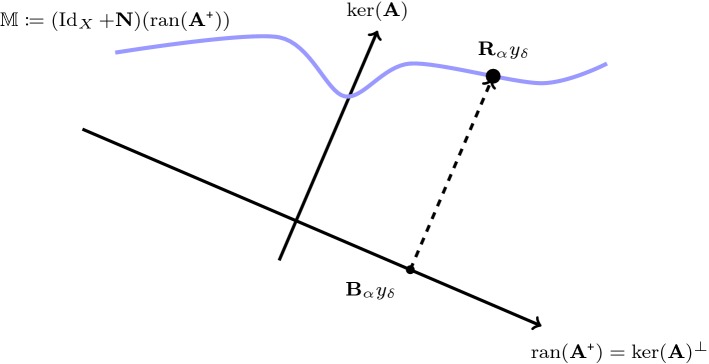
Regularization defined by a null space network. For a filter-based regularization method, we have $\mathbf {B}_\alpha y_{\delta }\in \ker (\mathbf {A})^\bot$. The regularized null space network $\mathbf {R}_\alpha = \mathbf {B}_\alpha + \mathbf {N}\circ \mathbf {B}_\alpha$ adds reasonable parts along the null space $\ker (\mathbf {A})$ to the standard regularization $\mathbf {B}_\alpha y_{\delta }$

The regularized null space networks analyzed in [21] take the form

2.3 $$\begin{aligned} \mathbf {R}_\alpha :=({\text {Id}}_X+\mathbf {N})\circ \mathbf {B}_\alpha \quad \text {for } \alpha >0 , \end{aligned}$$

where $(\mathbf {B}_\alpha )_{\alpha >0}$ is any classical regularization method and ${\text {Id}}_X+\mathbf {N}$ any null space network (for example, defined by a trained deep neural network). In [21], we have shown that (2.3) yields a regularization method with admissible set $\mathbb {M}:=({\text {Id}}_X + \mathbf {N})({\text {ran}}(\mathbf {A}^{\varvec{\texttt {+}}}))$. This approach is designed to find the null space component of the solution in a data-driven manner with a fixed neural network $\mathbf {N}$ independent of the regularization parameter $\alpha$ that works in the null space of $\mathbf {A}$; compare Fig. 3.

In this paper, we go one step further and consider a series of regularizing networks (RegNets) of the form $({\text {Id}}_X + \mathbf {N}_{\theta (\alpha )} )\circ \mathbf {B}_\alpha$ generalizing null space networks of the form (2.3). Here, $\mathbf {N}_{\theta (\alpha )}$ depends on $\alpha$ and is allowed to act in the orthogonal complement of the kernel $\ker (\mathbf {A})^\perp$. We give conditions under which this approach yields a regularization method with admissible set $\mathbb {M}$.

Allowing the network $\mathbf {N}_{\theta (\alpha )}$ to also act in $\ker (\mathbf {A})^\perp$ in particular is beneficial, if the forward operator $\mathbf {A}$ contains many small singular values. In this case, the network can learn components which are not sufficiently well-contained in the data. Note that in the limit $\alpha \rightarrow 0$, the regularization method $(\mathbf {B}_\alpha )_{\alpha >0}$ converges to $\mathbf {A}^{\varvec{\texttt {+}}}$ point-wise. Therefore, in the limit $\alpha \rightarrow 0$, the network is restricted to learn components in the null space of $\mathbf {A}$.

### Related Work

Recently, many works using deep neural networks to solve inverse problems have been published. These papers include two-stage approaches, where in a first step an initial reconstruction is done, followed by a deep neural network. Several network architectures, often based on the U-net architecture [18] and improvements of it [9, 24], have been used for this class of methods.

CNN-based methods that only modify the part of the reconstruction that is contained in the null space of the forward operator have been proposed in [16, 17]. In [21], we introduced regularized null space networks which are shown to lead a convergent regularization method. Recently, a related synthesis approach for learning the invisible frame coefficients for limited angle computed tomography has been proposed in [6].

Another possibility to improve reconstructions by deep learning is to replace certain operations in an iterative scheme by deep neural networks or use learned regularization functionals [1, 2, 8, 12, 15]. Further, a Bayesian framework has been proposed in [3, 4], where the posterior distribution of solutions is approximated by learned CNNs.

## Convergence and Convergence Rates of RegNets

In this section, we formally introduce the concept of RegNets, analyze their regularization properties and derive convergence rates.

Throughout the following, let $\mathbf {A}:\mathbb {X}\rightarrow \mathbb {Y}$ be a linear and bounded operator and ${\text {Id}}_X + \mathbf {N}:\mathbb {X}\rightarrow \mathbb {X}$ be a null space network, see Definition 3. Further, let $(\mathbf {B}_\alpha )_{\alpha >0}$ denote a classical filter-based regularization method, defined by the regularizing filter $(g_\alpha )_{\alpha >0}$, see Definition 2.

### Convergence

Let us first formally define a family of regularizing networks.

#### Definition 5

Let $(\mathbf {B}_\alpha )_{\alpha >0}$ be a classical filter-based regularization method. A family $(\mathbf {N}_{\theta (\alpha )} )_{\alpha >0}$ of Lipschitz continuous functions $\mathbf {N}_{\theta (\alpha )} :\mathbb {X}\rightarrow \mathbb {X}$ is called $((\mathbf {B}_\alpha )_{\alpha >0}, \mathbf {N})$-adapted if

- $$ \lim _{\alpha \rightarrow 0} \mathbf {N}_{\theta (\alpha )} (\mathbf {B}_\alpha \mathbf {A}z) = \mathbf {N}(z)$$ for all $$z\in {\text {ran}}(\mathbf {A}^{\varvec{\texttt {+}}})$$.
- The Lipschitz constants of $(\mathbf {N}_{\theta (\alpha )})_{\alpha >0}$ are bounded from above by some constant $L>0$.

For the following recall Definition 4 of a regularization method with admissible set $\mathbb {M}$. We will often use the notation $\mathbf {N}z :=\mathbf {N}(z)$. The following convergence results hold.

#### Theorem 3.1

(RegNets) Let $(\mathbf {B}_\alpha )_{\alpha >0}$ be a classical filter-based regularization method and $(\mathbf {N}_{\theta (\alpha )} )_{\alpha >0}$ be $((\mathbf {B}_\alpha )_{\alpha >0}, \mathbf {N})$-adapted. Then, the family

3.1 $$\begin{aligned} \mathbf {R}_\alpha (y_{\delta })=({\text {Id}}_X+\mathbf {N}_{\theta (\alpha )} )\mathbf {B}_\alpha (y_{\delta }), \end{aligned}$$

is a regularization method with admissible set

3.2 $$\begin{aligned} \mathbb {M}:=({\text {Id}}_X + \mathbf {N}) ( {\text {ran}}(\mathbf {A}^{\varvec{\texttt {+}}}) ) . \end{aligned}$$

We call $(\mathbf {R}_\alpha )_{\alpha >0}$ a regularizing family of networks (RegNets) adapted to $((\mathbf {B}_\alpha )_{\alpha >0}, \mathbf {N})$.

#### Proof

Let $x_{\alpha ,\delta }:=\mathbf {R}_\alpha (y_{\delta })=({\text {Id}}_X+\mathbf {N}_{\theta (\alpha )} )\mathbf {B}_\alpha (y_{\delta })$. Then, we have

3.3 $$\begin{aligned}&\Vert x-x_{\alpha ,\delta }\Vert \nonumber \\&\quad =\Vert \mathbf {B}_\alpha \mathbf {A}x+({\text {Id}}_X-\mathbf {B}_\alpha \mathbf {A})x-\mathbf {B}_\alpha y_{\delta }-\mathbf {N}_{\theta (\alpha )} \mathbf {B}_\alpha y_{\delta }\Vert \nonumber \\&\quad \le \ \Vert \mathbf {B}_\alpha (\mathbf {A}x-y_{\delta })\Vert +\Vert ({\text {Id}}_X-\mathbf {B}_\alpha \mathbf {A})x-\mathbf {N}_{\theta (\alpha )} \mathbf {B}_\alpha \mathbf {A}x\Vert \nonumber \\&\qquad +\Vert \mathbf {N}_{\theta (\alpha )} \mathbf {B}_\alpha \mathbf {A}x-\mathbf {N}_{\theta (\alpha )} \mathbf {B}_\alpha y_{\delta }\Vert \nonumber \\&\quad \le \ (1+L)\Vert \mathbf {B}_\alpha \Vert \delta +\Vert x-\mathbf {N}_{\theta (\alpha )} \mathbf {B}_\alpha \mathbf {A}x-\mathbf {B}_\alpha \mathbf {A}x\Vert .\nonumber \\ \end{aligned}$$

Assuming that $x=({\text {Id}}_X+\mathbf {N})z\in \mathbb {M}$ with $z\in {\text {ran}}(\mathbf {A}^{\varvec{\texttt {+}}})$, we get

$$\begin{aligned}&\Vert x- x_{\alpha ,\delta }\Vert \\&\quad \le (1+L)\Vert \mathbf {B}_\alpha \Vert \delta + \Vert z +\mathbf {N}z-\mathbf {N}_{\theta (\alpha )} \mathbf {B}_\alpha \mathbf {A}z-\mathbf {B}_\alpha \mathbf {A}z\Vert \\&\quad \le (1+L)\Vert \mathbf {B}_\alpha \Vert \delta + \Vert z-\mathbf {B}_\alpha \mathbf {A}z\Vert + \Vert \mathbf {N}z-\mathbf {N}_{\theta (\alpha )} \mathbf {B}_\alpha \mathbf {A}z\Vert . \end{aligned}$$

Eventually, we get $\lim _{\delta \rightarrow 0} \Vert x-x_{\alpha ,\delta }\Vert = 0$ since the first expression vanishes by assumption, the second because $(\mathbf {B}_\alpha )_{\alpha >0}$ is a regularization method and the last because of $(\mathbf {N}_{\theta (\alpha )} )_{\alpha >0}$ being $((\mathbf {B}_\alpha )_{\alpha >0}, \mathbf {N})$-adapted. $\square$

### Convergence Rates

In this section, we derive convergence rates for RegNets introduced in Sect. 3.1. To that end, we first introduce a distance function and define the qualification of a classical regularization method. The definition of the distance function is essentially motivated by [11].

#### Definition 6

(*Distance function*) For any numbers $\alpha , \rho , \mu >0$ and $x \in \mathbb {X}$, we define the distance function

3.4 $$\begin{aligned} d_\alpha (x; \rho , \mu ):= & {} \inf \{\Vert x-\mathbf {N}_{\theta (\alpha )} \mathbf {B}_\alpha \mathbf {A}x-(\mathbf {A}^*\mathbf {A})^\mu \omega \Vert \nonumber \\&\mid \omega \in \mathbb {X}\wedge \Vert \omega \Vert \le \rho \}. \end{aligned}$$

The qualification of a regularization method is a classical concept in regularization theory (see [7, Theorem 4.3]) and central for the derivation of convergence rates.

#### Definition 7

(*Qualification*) We say that a filter-based regularization $\mathbf {B}_\alpha :=g_\alpha (\mathbf {A}^*\mathbf {A})\mathbf {A}^*$ defined by the regularizing filter $(g_\alpha )_{\alpha >0}$ has qualification at last $\mu _0 \in (0,\infty )$ if there is a constant $C>0$ such that for all $\mu \in (0,\mu _0]$, we have

3.5 $$\begin{aligned}&\forall \alpha >0 :\sup \{\lambda ^\mu \left| 1-\lambda g_\alpha (\lambda ) \right| \nonumber \\&\quad \mid \lambda \in [0,||\mathbf {A}^*\mathbf {A}||]\} \le C \alpha ^\mu . \end{aligned}$$

The largest value $\mu _0$ such that (3.5) holds for all $\mu \in (0,\mu _0]$ is called the qualification of the regularization method $(\mathbf {B}_\alpha )_{\alpha >0}$ or the regularizing filter $(g_\alpha )_{\alpha >0}$ (taken as infinity if (3.5) holds for all $\mu > 0$).

Note that Tikhonov regularization has qualification $\mu _0=1$, and truncated SVD regularization has infinite qualification. Further, if $(\mathbf {B}_\alpha )_{\alpha >0}$ has qualification $\mu _0$, then (see [7])

3.6 $$\begin{aligned}&{\left\| ({\text {Id}}_X-\mathbf {B}_\alpha \mathbf {A})(\mathbf {A}^*\mathbf {A})^\mu \omega \right\| } \le C \rho \alpha ^\mu \end{aligned}$$

3.7 $$\begin{aligned}&\Vert \mathbf {A}({\text {Id}}_X - \mathbf {B}_\alpha \mathbf {A})(\mathbf {A}^*\mathbf {A})^\mu \omega \Vert \le C \rho \alpha ^{\mu +1/2} \end{aligned}$$

holds for $\mu \le \mu _0$, $\alpha >0$ and all $\omega \in \mathbb {X}$ with ${\left\| \omega \right\| } \le \rho$.

#### Lemma 1

Let $(\mathbf {R}_\alpha )_{\alpha >0}$ be a family of RegNets adapted to $((\mathbf {B}_\alpha )_{\alpha >0}, \mathbf {N})$ where $(\mathbf {B}_\alpha )_{\alpha >0}$ has qualification of order at least $\mu$. Then, for any $\alpha , \delta , \rho >0$ and $x \in \mathbb {X}$,

3.8 $$\begin{aligned}&\Vert \mathbf {R}_\alpha (y_{\delta })-x\Vert \le \delta (1+L)\Vert \mathbf {B}_\alpha \Vert \nonumber \\&\quad +C\rho \alpha ^\mu +d_\alpha (x; \rho , \mu ) + \Vert \mathbf {B}_\alpha \mathbf {A}\mathbf {N}_{\theta (\alpha )} \mathbf {B}_\alpha \mathbf {A}x\Vert , \end{aligned}$$

where $y_{\delta }\in \mathbb {Y}$ satisfies $\Vert \mathbf {A}x -y_{\delta }\Vert \le \delta$ and *C* is the constant from Definition 7.

#### Proof

As in the proof of Theorem 3.1, we have

3.9 $$\begin{aligned}&\Vert x-x_{\alpha ,\delta }\Vert \le (1+L)\Vert \mathbf {B}_\alpha \Vert \delta \nonumber \\&\quad + \underbrace{\Vert x-\mathbf {N}_{\theta (\alpha )} \mathbf {B}_\alpha \mathbf {A}x-\mathbf {B}_\alpha \mathbf {A}x\Vert }_{=:E_\alpha }. \end{aligned}$$

Further for all $\omega \in \mathbb {X}$ with $\Vert \omega \Vert \le \rho$, the term $E_\alpha$ can be estimated as

$$\begin{aligned} E_\alpha&\le {\left\| x-\mathbf {N}_{\theta (\alpha )} \mathbf {B}_\alpha \mathbf {A}x- \mathbf {B}_\alpha \mathbf {A}(x-\mathbf {N}_{\theta (\alpha )} \mathbf {B}_\alpha \mathbf {A}x)\right\| }\\&\quad +\Vert \mathbf {B}_\alpha \mathbf {A}\mathbf {N}_{\theta (\alpha )} \mathbf {B}_\alpha \mathbf {A}x\Vert \\&= \Vert ({\text {Id}}_X-\mathbf {B}_\alpha \mathbf {A})(x-\mathbf {N}_{\theta (\alpha )} \mathbf {B}_\alpha \mathbf {A}x)\Vert \\&\quad + \Vert \mathbf {B}_\alpha \mathbf {A}\mathbf {N}_{\theta (\alpha )} \mathbf {B}_\alpha \mathbf {A}x\Vert \\&\le \ \Vert ({\text {Id}}_X-\mathbf {B}_\alpha \mathbf {A})(\mathbf {A}^*\mathbf {A})^\mu \omega \Vert \\&\quad +\Vert ({\text {Id}}_X-\mathbf {B}_\alpha \mathbf {A})(x-\mathbf {N}_{\theta (\alpha )} \mathbf {B}_\alpha \mathbf {A}- (\mathbf {A}^*\mathbf {A})^\mu \omega \Vert \\&\quad + \Vert \mathbf {B}_\alpha \mathbf {A}\mathbf {N}_{\theta (\alpha )} \mathbf {B}_\alpha \mathbf {A}x\Vert \\&\le \Vert ({\text {Id}}_X-\mathbf {B}_\alpha \mathbf {A})(\mathbf {A}^*\mathbf {A})^\mu \omega \Vert \\&\quad +d_\alpha (x; \rho , \mu )+\Vert \mathbf {B}_\alpha \mathbf {A}\mathbf {N}_{\theta (\alpha )} \mathbf {B}_\alpha \mathbf {A}x\Vert . \end{aligned}$$

Because $(\mathbf {B}_\alpha )_{\alpha >0}$ has qualification of order $\mu$, we have

$$\begin{aligned} E_\alpha \le C\rho \alpha ^\mu +d_\alpha (x; \rho , \mu ) +\Vert \mathbf {B}_\alpha \mathbf {A}\mathbf {N}_{\theta (\alpha )} \mathbf {B}_\alpha \mathbf {A}x\Vert , \end{aligned}$$

which concludes the proof. $\square$

From Lemma 1, we obtain the following theorem providing convergence rates for families of RegNets.

#### Theorem 3.2

(Convergence rate) Let $(\mathbf {R}_\alpha )_{\alpha >0}$ be a family of RegNets adapted to $((\mathbf {B}_\alpha )_{\alpha >0}, \mathbf {N})$ for some classical regularization $(\mathbf {B}_\alpha )_{\alpha }$ and $\mathbb {M}$ defined by a null space network ${\text {Id}}_X + \mathbf {N}$. Further, assume that for a set $\mathbb {M}_{\rho ,\mu } \subseteq \mathbb {M}$, the following hold:

1. The parameter choice rule satisfies $\alpha \asymp \delta ^{\frac{2}{2\mu +1}}$.
2. For all $x \in \mathbb {M}_{\rho ,\mu }$ we have
  $$\begin{aligned} d_\alpha (x; \rho , \mu ) = {{\mathcal {O}}}(\alpha ^\mu ) \text { as } \alpha \rightarrow 0 \end{aligned}$$
3. For all $x \in \mathbb {M}_{\rho ,\mu }$ we have
  $$\begin{aligned} \Vert \mathbf {B}_\alpha \mathbf {A}\mathbf {N}_{\theta (\alpha )} \mathbf {B}_\alpha \mathbf {A}x\Vert ={{\mathcal {O}}}(\alpha ^\mu ) \text { as } \alpha \rightarrow 0 . \end{aligned}$$
4. $(\mathbf {B}_\alpha )_{\alpha >0}$ has qualification at least $\mu$.

Then for all $x\in \mathbb {M}_{\rho ,\mu }$, the following convergence rates result holds

3.10 $$\begin{aligned} \Vert \mathbf {R}_\alpha (y_{\delta })-x\Vert = {{\mathcal {O}}}(\delta ^{\frac{2\mu }{2\mu +1}}) \text { as } \alpha \rightarrow 0 . \end{aligned}$$

#### Proof

The assertion follows from Lemma 1. $\square$

In the following section, we will give three examples of regularization methods that arise as special cases of our results given above. In particular, we give a data-driven extension of SVD regularization where the assumptions of Theorem 3.2 are satisfied.

## Special Cases

In this section, we demonstrate that our theory recovers known existing results as special cases and demonstrate how to derive novel data-driven regularization methods. In particular, we show that any classical regularization method, regularization by null space networks and a deep learning variant of truncated SVD fit within our framework introduced in Sect. 3.

### Classical Filter-Based Regularization

Classical Tikhonov regularization is a special case of the regularization method defined in Theorem 3.1 with

$$\begin{aligned}&\mathbf {B}_\alpha =(\mathbf {A}^*\mathbf {A}+\alpha {\text {Id}}_X)^{-1}\mathbf {A}^*\\&\mathbf {N}_{\theta (\alpha )} =0 . \end{aligned}$$

In this case, the distance function

$$\begin{aligned} d_\alpha (x; \rho , \mu )=\inf \{\Vert x-(\mathbf {A}^*\mathbf {A})^\mu \omega \Vert \mid \omega \in \mathbb {X}\wedge \Vert \omega \Vert \le \rho \} \end{aligned}$$

is independent of $\alpha$, and therefore satisfies $d_\alpha (x; \rho , \mu ) = {\mathcal {O}}(\alpha ^\mu )$ if and only if $d_\alpha (x; \rho , \mu )=0$. This in turn is equivalent to

$$\begin{aligned} x\in \{(\mathbf {A}^*\mathbf {A})^\mu \omega \mid \omega \in \mathbb {X}\wedge \Vert \omega \Vert \le \rho \} , \end{aligned}$$

which is the classical source condition for the convergence rate $\Vert x-x_{\alpha ,\delta }\Vert ={\mathcal {O}}(\delta ^{\frac{2\mu }{2\mu +1}})$ as $\delta \rightarrow 0$.

Clearly, the above considerations equally apply to any filter-based regularization method including iterative Tikhonov regularization, truncated SVD and the Landweber iteration. We conclude that Theorem 3.2 contains classical convergence rates results for classical regularization methods as special cases.

### Regularized Null Space Networks

In the case of regularized null space networks, we take $(\mathbf {B}_\alpha )_{\alpha >0}$ as a filter-based regularization method and $\mathbf {N}_{\theta (\alpha )} = \mathbf {N}$ for some null space network ${\text {Id}}_X + \mathbf {N}$. In the following theorem, we derive a decay rate of the distance function on the source set

$$\begin{aligned}&\mathbb {X}_{\mu ,\rho } :=\{({\text {Id}}_X+\mathbf {N})(\mathbf {A}^*\mathbf {A})^\mu \omega \mid \omega \in \mathbb {X}\\&\quad \text { and } \Vert \omega \Vert \le \rho \} \end{aligned}$$

in the special case where the regularizing networks are given by a regularized null space network.

For regularized null space networks, in [21, Theorem 2.8], we derive the convergence rate $\Vert \mathbf {R}_\alpha (y_{\delta })-x\Vert = {\mathcal {O}}(\delta ^{\frac{2\mu }{2\mu +1}})$ for $x \in \mathbb {X}_{\mu ,\rho }$ and $\alpha \asymp \delta ^{\frac{2}{2\mu +1}}$. The following theorem shows that [21, Theorem 2.8] is a special case of Theorem 3.2. In this sense, the results of the current paper are indeed an extension of [21].

#### Theorem 4.1

(Convergence rates for regularized null space networks) Let ${\text {Id}}_X + \mathbf {N}:\mathbb {X}\rightarrow \mathbb {X}$ be a null space network and take $\mathbf {N}_{\theta (\alpha )} =\mathbf {N}$ for all $\alpha >0$. Further, let $(\mathbf {B}_\alpha )_{\alpha >0}$ be a classical regularization method with qualification at least $\mu$ that satisfies $\mathbf {N}\mathbf {B}_\alpha (0)=0$. Then, we have

4.1 $$\begin{aligned} d_\alpha (x; \rho , \mu )={\mathcal {O}}(\alpha ^\mu ) \quad \text { for all x } \in \mathbb {X}_{\mu ,\rho } . \end{aligned}$$

In particular, if $(\mathbf {B}_\alpha )_{\alpha >0}$ has qualification $\mu$, then the parameter choice $\alpha \asymp \delta ^{2/(2\mu +1)}$ gives the convergence rate $\Vert \mathbf {R}_\alpha (y_{\delta })-x\Vert = {\mathcal {O}}(\delta ^{2\mu /(2\mu +1)})$ for $x \in \mathbb {X}_{\rho , \mu }$.

#### Proof

For $x\in \mathbb {X}_{\mu ,\rho }$, we have

$$\begin{aligned}&\Vert x-\mathbf {N}\mathbf {B}_\alpha \mathbf {A}x-(\mathbf {A}^*\mathbf {A})^\mu \omega \Vert \\&\quad = \Vert \mathbf {N}(\mathbf {A}^*\mathbf {A})^\mu \omega - \mathbf {N}\mathbf {B}_\alpha \mathbf {A}(\mathbf {A}^*\mathbf {A})^\mu \omega \\&\qquad -\mathbf {N}\mathbf {B}_\alpha \mathbf {A}\mathbf {N}(\mathbf {A}^*\mathbf {A})^\mu \omega \Vert \\&\quad =\Vert \mathbf {N}(\mathbf {A}^*\mathbf {A})^\mu \omega - \mathbf {N}\mathbf {B}_\alpha \mathbf {A}(\mathbf {A}^*\mathbf {A})^\mu \omega \Vert \\&\quad \le L \Vert ({\text {Id}}_X-\mathbf {B}_\alpha \mathbf {A})(\mathbf {A}^*\mathbf {A})^\mu \omega \Vert \\&\quad \le L C\alpha ^\mu . \end{aligned}$$

Here, *L* denotes the Lipschitz constant of $\mathbf {N}$ and *C* is some constant depending on the regularization $(\mathbf {B}_\alpha )_{\alpha >0}$. $\square$

### Data-Driven Continued SVD

For the following, assume that $\mathbf {A}$ admits a singular value decomposition

$$\begin{aligned} \left( (u_n)_{n\in \mathbb {N}},(v_n)_{n\in \mathbb {N}}, (\sigma _n)_{n\in \mathbb {N}}\right) , \end{aligned}$$

where $(u_n)_{n\in \mathbb {N}}$ and $(v_n)_{n\in \mathbb {N}}$ are orthonormal systems in $\mathbb {X}$ and $\mathbb {Y}$, respectively, and $\sigma _n$ are positive numbers such that for all $x\in \mathbb {X}$

4.2 $$\begin{aligned} \mathbf {A}x= \sum _{n\in \mathbb {N}} \sigma _n \langle u_n,x\rangle v_n. \end{aligned}$$

The regularization method corresponding to the regularizing filter given in (2.2) yields to the truncated SVD given by

4.3 $$\begin{aligned} \mathbf {B}_\alpha (y) = \sum _{\sigma _n^2\ge \alpha } \frac{1}{\sigma _n}\langle y, v_n \rangle u_n. \end{aligned}$$

The truncated SVD only recovers signal components corresponding to sufficiently large singular values of $\mathbf {A}$ and sets the other components to zero. It seems reasonable to train a network that extends the coefficients with nonzero values, and therefore can better approximate non-smooth functions.

To achieve a learned data extension, we consider a family of regularizing networks of the form (3.1)

4.4 $$\begin{aligned} \mathbf {R}_\alpha (y_{\delta })&:=({\text {Id}}_X+\mathbf {N}_{\theta (\alpha )} )\mathbf {B}_\alpha (y_{\delta })\nonumber \\&= ({\text {Id}}_X+\mathbf {N}_{\theta (\alpha )} ) \sum _{\sigma _n^2\ge \alpha } \frac{1}{\sigma _n}\langle y_{\delta }, v_n \rangle u_n \end{aligned}$$

4.5 $$\begin{aligned} \mathbf {N}_{\theta (\alpha )} (z)&:=({\text {Id}}_X-\mathbf {B}_\alpha \mathbf {A}) \mathbf {U}_{\theta (\alpha )} (z)\nonumber \\&= \sum _{\sigma _n^2 < \alpha } \langle \mathbf {U}_{\theta (\alpha )} z, u_n \rangle u_n . \end{aligned}$$

For the data-driven continued SVD (4.4), (4.5), the following convergence rates result holds.

#### Theorem 4.2

(Convergence rates for data-driven continued SVD) Let $(\mathbf {R}_\alpha )_{\alpha >0}$ be defined by (4.4), (4.5) and adapted to $((\mathbf {B}_\alpha )_{\alpha >0}, \mathbf {N})$, where $(\mathbf {B}_\alpha )_{\alpha >0}$ is given by truncated SVD and $\mathbb {M}$ is defined by (3.2) for some null space network ${\text {Id}}_X + \mathbf {N}$. Moreover, assume that $d_\alpha (x; \rho , \mu ) = {\mathcal {O}}(\alpha ^\mu )$ for all $x \in \mathbb {M}_{\rho ,\mu }$ in some set $\mathbb {M}_{\rho ,\mu } \subseteq \mathbb {M}$. Then, provided that $\alpha \asymp \delta ^{\frac{2}{2\mu +1}}$, for all $x\in \mathbb {M}_{\rho ,\mu }$, we have

4.6 $$\begin{aligned} \Vert \mathbf {R}_\alpha (y_{\delta })-x\Vert = {\mathcal {O}}(\delta ^{\frac{2\mu }{2\mu +1}}) \text { as } \alpha \rightarrow 0 . \end{aligned}$$

#### Proof

We apply Theorem 3.2 and for that purpose verify (A1)–(A4). Items (A1) and (A2) are satisfied according to the made assumptions. Moreover, we have

$$\begin{aligned} {\text {ran}}( ({\text {Id}}_X-\mathbf {B}_\alpha \mathbf {A}) \mathbf {U}_{\theta (\alpha )} )\subseteq {\text {span}}\{u_i \mid \sigma _i^2<\alpha \} . \end{aligned}$$

Then for $x\in \mathbb {X}$ and all $\alpha$, $\Vert \mathbf {B}_\alpha \mathbf {A}\mathbf {N}_{\theta (\alpha )} \mathbf {B}_\alpha \mathbf {A}x\Vert$ vanishes, and therefore (A3) is satisfied. Finally, it is well-known that truncated SVD has infinite qualification [7, Example 4.8], which gives Assumption (A4) in Theorem 3.2 and concludes the proof. $\square$

The networks $\mathbf {N}_{\theta (\alpha )}$ map the truncated SVD reconstruction $\mathbf {B}_\alpha (y_{\delta })$ lying in the space spanned by the reliable basis elements (corresponding to sufficiently large singular values of the operator $\mathbf {A}$) to coefficients unreliably predicted by $\mathbf {A}$. Hence, opposed to truncated SVD, $\mathbf {R}_\alpha$ is some form of continued SVD, where the extension of the unreliable coefficients is learned from the reliable ones in a data-driven manner.

Opposed to the two previous examples, for the data-driven continued SVD, we do not have a simple and explicit characterization for the sets $\mathbb {M}_{\rho ,\mu }$ in Theorem 4.2. These sets crucially depend on the nature of the networks $\mathbf {N}_{\theta (\alpha )}$, the used training data and training procedure. Investigating and characterizing these sets in particular situations will be subject of future research.

Another natural example is the case where classical Tikhonov regularization $\mathbf {B}_\alpha =(\mathbf {A}^*\mathbf {A}+\alpha {\text {Id}}_X)^{-1}\mathbf {A}^*$ is used to define a RegNet $(\mathbf {R}_\alpha )_\alpha$ of the form (3.1). Also in this example, Theorem 3.1 gives convergence of $(\mathbf {R}_\alpha )_\alpha$ under the assumption that $(\mathbf {N}_{\theta (\alpha )})_{\alpha >0}$ is adapted to $((\mathbf {B}_\alpha )_{\alpha >0}, \mathbf {N})$. However, for Tikhonov regularization, we are currently not able to verify (A3) under natural assumptions, required for the convergence rates results. Investigating convergence rates for the combination of Tikhonov regularization or other regularization methods with a learned component will be investigated in future research.

## Numerical Example

In this section, we consider the inverse problem $g = \mathcal {R}(f)$, where $\mathcal {R}$ is an undersampled Radon transform. For that purpose, we compare classical truncated SVD, the data-driven extended SVD and the null space approach of [21]. Similar results are presented in [22] for the limited data problem of photoacoustic tomography.

### Discretization

We discretize the Radon transform $\mathcal {R}$ by using radial basis functions. For a phantom $f:\mathbb {R}^2 \rightarrow \mathbb {R}$ supported in the domain $[-1,1]^2$, we make the basis function ansatz

5.1 $$\begin{aligned} f(x) = \sum _{i=1}^{N^2} c_i \varphi _i(x), \end{aligned}$$

for coefficients $c_i\in \mathbb {R}$ and $\varphi _i(x) = \varphi (x-x_i)$, where $x_i$ are arranged on a Cartesian grid on $[-1,1]^2$ and $\varphi :\mathbb {R}^2\rightarrow \mathbb {R}$ is the Kaiser–Bessel function given by

5.2 $$\begin{aligned} \varphi (x) = {\left\{ \begin{array}{ll} \frac{I_0\left( (\rho \sqrt{1-(\Vert x\Vert /a)^2)}\right) }{I_0(\rho )} \quad &{} {\left\| x\right\| }\le a ,\\ 0 &{} \text {otherwise} . \end{array}\right. } \end{aligned}$$

Here, $I_0$ denotes the modified first kind Bessel function, and the parameters controlling the shape and support are chosen $\rho =7$ and $a=0.055$ (around 4 pixels in the images shown), respectively. We take advantage of the fact that for Kaiser–Bessel functions, the Radon transform is known analytically [14].

For our simulations, we evaluate the Radon transform at $N_\theta =30$ equidistant angles in $\theta _k:={(k-1)\pi }/{N_\theta }$ and $N_s=200$ equidistant distances to the origin in the interval $[-3/2,3/2]$. Further, we use a total number of $N^2=128^2$ basis function to approximate the unknown density *f*. Then, the discrete forward operator $\mathbf {A}\in \mathbb {R}^{N_s N_\theta \times N^2}$ is defined by $\mathbf {A}_{N_s(n-1)+j,i} = \mathcal {R}(\varphi _i) (s_n,t_j)$. This results in the following inverse problem for the coefficients of the phantom

5.3 $$\begin{aligned} \text {Recover } c\in \mathbb {R}^{N^2} \text { from data}\;\; y= \mathbf {A}c+\xi . \end{aligned}$$

Here, the vector $\xi \in \mathbb {R}^{N^2}$ models the error in the data.

For our choice of $N_\theta$, the Radon transform is highly undersampled, and (5.3) is ill-conditioned. In the following, we consider the problem of recovering *c*, since the function *f* can be reconstructed by evaluating (5.1). Note that $\varphi _i$ are translated versions of a fixed basis function with centers on a Cartesian grid. Therefore, we can naturally arrange the coefficients $c\in \mathbb {R}^{N^2}$ as an $N\times N$ image. This image representation will be used for visualization and for the inputs of the regularizing networks.

### Used Regularization Methods

Let $\mathbf {A}= U \Sigma V^\intercal$ be the singular value decomposition of the discrete forward operator. We denote by $(u_n)_{n=1}^{N_tN_\theta }$ and $(v_n)_{n=1}^{N^2}$ the columns of *U* and *U*, respectively, and by $\sigma _1\ge \sigma _2\ge \ldots \ge \sigma _{N_sN_\theta }$ the singular values. Singular vectors $u_n$ with vanishing singular values correspond to components of the null space $\ker (\mathbf {A})$.

- The truncated SVD $(\mathbf {B}_\alpha )_{\alpha >0}$ is then given by
  5.4 $$\begin{aligned} \mathbf {B}_\alpha (y) = \sum _{\sigma _n^2\ge \alpha } \frac{1}{\sigma _n}\langle y,v_n\rangle u_n \quad \text {for } y\in \mathbb {R}^{N_sN_\theta } . \end{aligned}$$
- The data-driven continued SVD (see (4.4), (4.5)) is of the form
  5.5 $$\begin{aligned} \mathbf {R}_\alpha (y) = \mathbf {B}_\alpha (y) + \sum _{\sigma _n^2<\alpha }\langle \mathbf {U}_{\theta (\alpha )}(\mathbf {B}_\alpha y),u_n\rangle u_n , \end{aligned}$$
  where $\mathbf {U}_{\theta (\alpha )}:\mathbb {R}^{N^2}\rightarrow \mathbb {R}^{N^2}$ is a neural network that operates on elements of $\mathbb {R}^{N^2}$ as $N\times N$ images, subsequently followed by the projection onto the singular vectors corresponding to the truncated singular values. We use the same U-net architecture as described in [5] (without residual connection) for $\mathbf {U}_{\theta (\alpha )}$. Note that the network does not affect the non-vanishing coefficients of the truncated SVD, which means that $\mathbf {R}_\alpha$ and $\mathbf {B}_\alpha$ reconstruct the same low-frequency parts.
- Additionally, we apply the regularized null space network of [21] which with the help of the SVD can be evaluated by
  5.6 $$\begin{aligned} \mathbf {R}_{\alpha }^0 (y) = \mathbf {B}_\alpha (y) + \sum _{\sigma _n^2 = 0}\langle \mathbf {U}_{\theta (\alpha )}^0(\mathbf {B}_\alpha y),u_n\rangle v_n . \end{aligned}$$
  For the neural network $\mathbf {U}_{\theta (\alpha )}$, we use again the U-net architecture as described as above. Opposed to (5.5), the null space networks only add components of the kernel $\ker (\mathbf {A})$ to $\mathbf {B}_\alpha$.

Note that the implemented regularization methods fit in the general framework of RegNets, see Sect. 4. In particular, for all methods, we have convergence as $\delta \rightarrow 0$. For the data-driven continued SVD (5.5), this convergence result requires that there is some network $\mathbf {U}:\mathbb {X}\rightarrow \mathbb {X}$ such that for all $x\in {\text {ran}}(\mathbf {A}^{\varvec{\texttt {+}}})$, we have

$$\begin{aligned} \lim _{\alpha \rightarrow 0} \sum _{\sigma _n^2<\alpha }\langle \mathbf {U}_{\theta (\alpha )} (\mathbf {P}_\alpha x), u_n\rangle u_n = \sum _{\sigma _n = 0 }\langle \mathbf {U}x,u_n\rangle u_n , \end{aligned}$$

where $\mathbf {P}_\alpha (x) :=\sum _{\sigma _n^2\ge \alpha } \langle x,u_n\rangle u_n$. We think that this convergence (at least on a reasonable subset of ${\text {ran}}(\mathbf {A}^{\varvec{\texttt {+}}})$) is reasonable using the same training strategy (5.7). Further, theoretical and practical research, however, is required for rigorously analyzing this issue.

### Network Training and Reconstruction Results

The regularizing networks $\mathbf {R}_\alpha$ and $\mathbf {R}_\alpha ^0$ were trained for different regularization parameters $\alpha$. Our training set consists of 1000 Shepp–Logan-type phantoms $c^{(k)}$ for $k = 1, \dots ,1000$ as ground truth and the corresponding regularized reconstructions $\mathbf {B}_\alpha y^{(k)}$ where the data $y^{(k)}=\mathbf {A}c^{(k)}$ were simulated with the discrete forward operator $\mathbf {A}$. We trained the network $\mathbf {R}_\alpha$ (and likewise $\mathbf {R}_\alpha ^0$) by minimizing the mean absolute error (MAE)

**Fig. 4:**
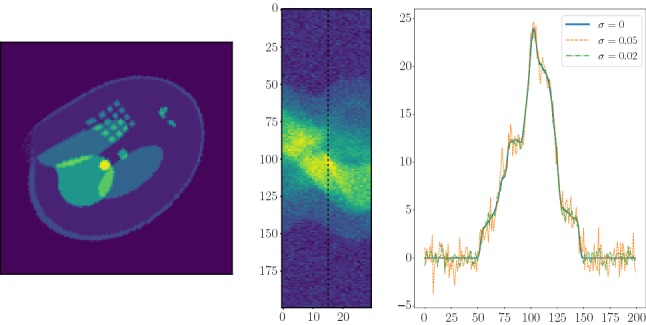
Right: True phantom from the test set. Middle: Simulated sparse Radon data $\mathbf {A}c+\delta \xi$ for $\mathbb {N}_\theta = 30$ directions, where $\xi _j\sim {\left\| \mathbf {A}c\right\| }_\infty \mathcal {N}(0,1)$ with $\delta =0.05$. Left: Cross section of the data for the 15th sensor directions for different noise levels

5.7 $$\begin{aligned} \frac{1}{1000}\sum _{k=1}^{1000} \Vert c^{(k)}-\mathbf {R}_\alpha (y^{(k)})\Vert _1, \end{aligned}$$

with the stochastic gradient descent (SGD) algorithm. The learning rate was set to 0.05 and the momentum parameter to 0.99. To evaluate the proposed regularizing networks, we generated 250 phantoms for testing (see Fig. 4 for an example from the test set).

**Fig. 5:**
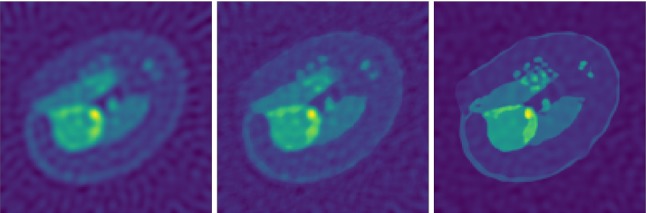
Reconstructions for low noise levels $(\delta =0.02)$. Left: Truncated SVD. Middle: Null space network. Right: Reconstruction with continued SVD

**Fig. 6:**
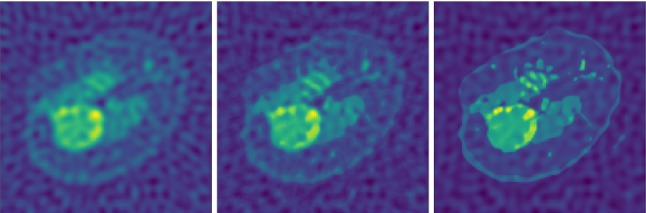
Reconstructions for higher noise levels $(\delta =0.05)$. Left: Truncated SVD. Middle: Null space network. Right: Reconstruction with continued SVD

We trained the networks $\mathbf {R}_\alpha$ and $\mathbf {R}_\alpha ^0$ for 15 different values of the regularization parameter $\alpha$ the same way using noise-free data minimizing (5.7) for $\mathbf {R}_\alpha$ and $\mathbf {N}_\alpha$, respectively. For the reconstructed images shown in Fig. 5 and Fig. 6, we took ten different images with corresponding data $y^{(k)} = \mathbf {A}c^{(k)} + \delta \xi ^{(k)}$ with noise level of $\delta =0.05$, where $\xi ^{(k)} \sim \Vert \mathbf {A}c^{(k)}\Vert _\infty \mathcal {N}(0,1)$. Then, we chose the regularization parameter with minimal mean squared error, averaged over the ten sample images. The resulting regularization parameter was $\alpha =1$ (which equals to taking the 796 biggest singular values).

**Fig. 7:**
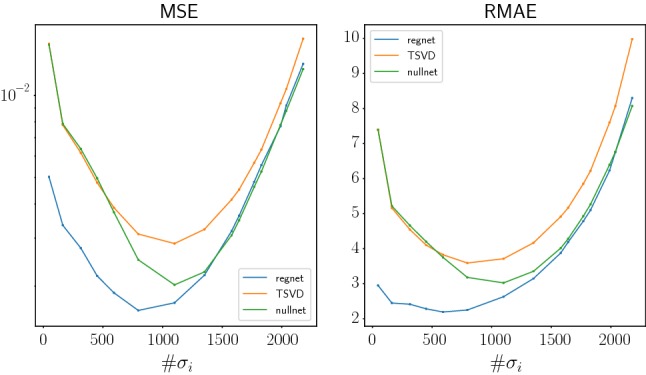
Mean Errors for the test images using different error measures. The *x*-axis shows the number of used singular values. The noise level is $\delta =0.02$

**Fig. 8:**
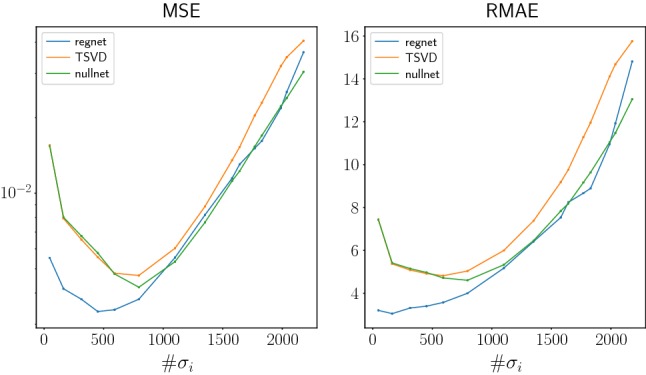
Mean Errors for the test images using different error measures. The *x*-axis shows the number of used singular values. The noise level is $\delta =0.05$

For quantitative evaluation of the different approaches, we calculated the mean errors for all 250 test images and all regularization parameters using the mean squared error (MSE) and the mean absolute error (MAE). All images were rescaled to have values in [0, 1] before calculating the error. The resulting error curves depending on the regularization parameter $\alpha$ (respectively, the number of used singular values) are shown in Figs. 7 and 8.

### Discussion

One can see that our proposed approach (data-driven continued SVD) in both cases outperforms the truncated SVD and the null space network; see Figs. 5 and 6. The better performance is also shown in Figs. 7 and 8, where the reconstruction errors are shown for varying regularization parameter (the number of used singular values). The data-driven continued SVD yields the smallest reconstruction errors followed by the null space network and the truncated SVD.

Interestingly, in these figures, one also observes a shift to the left of the error curve for the methods with learned components compared to plain truncated SVD. This can be explained as follows. The continued SVD and the null space network preserve the singular components corresponding to large singular values. Further, the reconstruction error corresponding to the truncated components is reduced by applying the trained network, and therefore the overall error becomes reduced compared to the other two methods. We conclude that partially learned methods need less singular values to achieve accurate results. This effect is even larger for the learned SVD than for the null space network. This explains the improved performance of the learned SVD and the shift to the left in Figs. 7 and 8.

There exists a variety of recently proposed deep learning-based methods for solving inverse problems, and in particular, for limited data problems in image reconstruction. Because the main contribution of our work is the theoretical analysis, we do not make the attempt here to numerically compare our method with other deep learning-based methods, for which no comparable theory is available. One advantage of our approach that we expect is the better generalization to data different from the training data. Numerical studies investigating such issues are subject of future research.

### Extensions

The probably most established deep learning approach to image reconstruction is to apply a two-step reconstruction network $\mathbf {R}_\mathrm{FBP} :=({\text {Id}}+ \mathbf {U}_{\theta } ) \circ \mathbf {B}_\mathrm{FBP}$ where $\mathbf {B}_\mathrm{FBP}$ denotes the filtered backprojection operator and $({\text {Id}}+ \mathbf {U}_{\theta })$ is a trained residual network. The FBP $\mathbf {B}_\mathrm{FBP}$ can been seen as a regularization method in the case of full data. In the case of limited data, this is not the case, and therefore it does not fully fit into the framework of our theory. Analyzing such more general situations opens an interesting line of research that we aim to address in future work.

Another interesting generalization of our results is the extension to regularization also from left and from the right. In this case, the reconstruction networks have the form

$$\begin{aligned} \mathbf {R}_{\alpha , \beta } (y) :=\mathbf {B}_{\beta }^{(1)} ({\text {Id}}+ \mathbf {N}_{\theta (\alpha , \beta )} ) \circ \mathbf {B}_{\alpha }^{(0)} \circ (y) , \end{aligned}$$

for regularization methods $(\mathbf {B}_{\alpha }^{(0)})_\alpha$, $(\mathbf {B}_{\beta }^{(1)})_\beta$ and networks $\mathbf {N}_{\theta (\alpha , \beta )}$. Extensions are even possible using cascades of network, which would have similarity with iterative and variational networks [2, 12] and cascades of networks [13, 20]. We expect that our results can be extended to such more general situations.

## Conclusion

In this paper, we introduced the concept of regularizing families of networks (RegNets), which are sequences of deep CNNs. The trained components of the networks, as well as the classical parts, are allowed to depend on the regularization parameter, and it is shown that under certain assumptions, this approach yields a convergent regularization method. We also derived convergence rates under the assumption that the solution lies in a source set that is different from the classical source sets. Examples were given, where the assumptions are satisfied. It has been shown that the new framework recovers results for classical regularization as special cases as well as data-driven improvements of classical regularization. Such data-driven regularization methods can give better results in practice than classical regularization methods which only use handcrafted prior information.

As a numerical example, we investigated a sparse sampling problem for the Radon transform. As regularization method, we took the truncated SVD and its data-driven counterparts, the null space network and the continued SVD. Numerical results clearly demonstrate that the continued SVD outperforms classical SVD as well as the null space network. Future work will be done to test the proposed regularizing networks on further ill-posed inverse problems and compare it with various other regularization methods. A detailed numerical comparison of our method with other deep learning methods is subject of future research. This will reveal the theoretical advantage of our method that it actually has improved generalizability.
